# Relationship between anxiety and depressive symptoms and social support in patients with atrial fibrillation

**DOI:** 10.1590/0034-7167-2025-0146

**Published:** 2026-07-31

**Authors:** Amanda da Silva Monteiro, Gustavo Carvalho de Lima Queiroz, Rayana Pereira Feitosa, José Arthur Guimarães dos Santos, João Victor Batista Cabral, Alcides Viana de Lima, Mailson Marques de Sousa

**Affiliations:** I Universidade Federal da Paraíba. João Pessoa, Paraíba, Brazil; II Universidade Federal do Rio Grande do Norte. Santa Cruz, Rio Grande do Norte, Brazil

**Keywords:** Atrial Fibrillation, Arrhythmias, Anxiety, Depression, Social Support., Fibrilación Atrial, Arritmias Cardíacas, Ansiedad, Depresión, Apoyo Social.

## Abstract

**Objectives:**

to analyze the relationship between anxiety and depressive symptoms and social support in patients with atrial fibrillation.

**Methods:**

a descriptive, cross-sectional, correlational study was conducted with 102 participants. Data were collected using the Hospital Anxiety and Depression Scale and the Medical Outcomes Study Social Support Survey. Descriptive statistics and Spearman’s rank correlation were used to analyze the data.

**Results:**

the median anxiety score was 8.0 (interquartile range [IQR] 7-11), and the median depression score was 6.0 (IQR 3-10). Anxiety symptoms were identified in 60.8% of participants and depressive symptoms in 38.2%. Inverse correlations were observed between depressive symptoms and affectionate support, emotional/informational support, and positive social interaction. No associations were found between anxiety symptoms and social support.

**Conclusions:**

higher levels of affectionate and emotional/informational support and positive social interaction were associated with lower depressive symptom scores.

## RESUMEN

**Objetivos:**

analizar la relación entre síntomas de ansiedad y depresión y apoyo social en pacientes con fibrilación auricular.

**Métodos:**

estudio descriptivo, transversal y correlacional con 102 participantes. Los datos se recogieron con la Escala de Ansiedad y Depresión Hospitalaria y la Escala de Apoyo Social. Se utilizaron estadística descriptiva y correlación de Spearman para el análisis de los datos.

**Resultados:**

la mediana de ansiedad fue 8,0 (RIC 7-11) y la de depresión 6,0 (RIC 3-10). Se identificaron síntomas de ansiedad en 60,8% y de depresión en 38,2% de los participantes. Se observó correlación inversa entre apoyo afectivo, apoyo emocional/informacional e interacción social positiva y síntomas depresivos. No se hallaron asociaciones entre síntomas de ansiedad y apoyo social.

**Conclusiones:**

niveles más altos de apoyo afectivo, emocional/informacional e interacción social positiva se asocian con puntuaciones más bajas de síntomas depresivos.

## INTRODUCTION

Atrial fibrillation (AF) is the most common supraventricular tachyarrhythmia encountered in clinical practice. It is characterized by disorganized atrial electrical activity, with uncoordinated electrical impulses and loss of effective atrial contraction^(1)^.

The incidence of AF has increased by 13% over the past 20 years, with 5 million new cases annually, largely driven by population aging, therapeutic advances, and improved survival among people with chronic diseases^(2,3)^. An estimated 59 million people worldwide live with AF^(4)^. In Brazil, data from the First Brazilian Cardiovascular Registry of Atrial Fibrillation (RECALL) indicate that the condition affects about 1.5 million individuals^(5)^. It is more prevalent among older adults^(1)^ and is considered a public health problem.

Managing patients diagnosed with AF involves lifestyle changes, symptom recognition, management of comorbidities, and adherence to pharmacological therapy to mitigate adverse events such as stroke, heart failure (HF), depression, and increased costs for health services^(3)^.

Anxiety and depressive symptoms are common among people with AF^(1,3)^. These psychological conditions are associated with poorer perceived quality of life (QoL) and may influence clinical outcomes, creating a vicious cycle that often culminates in psychological distress, limitations in activities of daily living, poor treatment adherence, and an increase in potentially avoidable hospitalizations^(6,7)^. In this context, social support is a key strategy in the management of these individuals, as it helps patients cope emotionally with the disease, supports treatment adherence, and promotes greater physical and psychological well-being^(1)^.

Social support is conceptualized as the availability of support from others, especially family members, friends, and health professionals^(8)^. This concept encompasses individuals’ level of social integration, as well as the extent to which interpersonal relationships can help them in times of crisis or adjustment. Thus, social support plays a protective role in coping with AF, helping patients adopt and maintain healthy lifestyle behaviors^(3)^.

Recent European Society of Cardiology guidelines recommend that assessment of AF-related symptoms such as anxiety and depression be incorporated into clinical practice as part of comprehensive care^(2,6)^. Therefore, investigating the relationship between anxiety, depression, and social support in patients with AF is particularly relevant to improving nurses’ clinical practice, since they are responsible for follow-up and for implementing longitudinal care. They also need to develop competencies to identify and manage risk factors that may contribute to AF progression.

In Latin America, research on this topic is still limited. To date, no studies have been identified in the Northeast Region of Brazil, which justifies conducting this and future investigations. Accordingly, this study addressed the following question: What is the relationship between anxiety and depressive symptoms and social support in patients with AF?

## OBJECTIVES

To analyze the relationship between anxiety and depressive symptoms and social support in patients with atrial fibrillation.

## METHODS

### Ethical aspects

This study was conducted in accordance with the ethical principles of Resolution No. 466/2012 of the Brazilian National Health Council and was approved by the Research Ethics Committee. Participants provided written informed consent by signing two copies of the informed consent form.

### Study design, period, and setting

This was a descriptive, cross-sectional, correlational study. The Strengthening the Reporting of Observational Studies in Epidemiology (STROBE) recommendations were followed. The study was conducted at the cardiology outpatient clinic of Lauro Wanderley University Hospital (HULW), located in the city of João Pessoa, Paraíba, Brazil, between September 2023 and June 2024. This large teaching hospital is affiliated with the Federal University of Paraíba (UFPB) and the Brazilian Unified Health System (*Sistema Único de Saúde*, SUS), and managed by the Brazilian Hospital Services Company (Empresa Brasileira de Serviços Hospitalares, EBSERH).

### Population and sample; inclusion and exclusion criteria

The study population comprised 135 patients with AF under outpatient follow-up at the institution. Sample size was calculated using the OpenEpi software program (version 3.01), based on data from a previous study^(9)^ and a previously reported prevalence of 34.9% for anxiety symptoms, with a 95% confidence level and a 5% margin of error. Thus, the minimum sample size was set at 98 participants.

We first reviewed the medical records of patients under outpatient follow-up at the institution to identify potentially eligible participants who met the inclusion criteria. After this screening, patients were invited to take part in the study.

We included individuals with a clinical diagnosis of AF, aged ≥18 years, and on chronic oral anticoagulation. We excluded patients who were undergoing treatment for anxiety or depression or taking anxiolytic and/or antidepressant medications, as well as those with cognitive impairment documented in their medical records.

### Study protocol

Two trained undergraduate nursing students collected data through individual interviews conducted in a private setting, with an average duration of 40 minutes, using the three instruments described below.

#### 1. Sociodemographic and clinical characterization form

This instrument was developed by the authors and included the following variables: sex (male or female); age (years); place of residence (João Pessoa, Paraíba, or other municipalities); skin color (White, Brown, Black, or Indigenous); educational attainment (years of completed education); employment status (employed or not employed); family income (in Brazilian reais); marital status (never married, common-law marriage/married, widowed, divorced/separated); comorbidities associated with AF; current pharmacological therapy; weekly anticoagulant dose (mg); duration of treatment (years); most recent International Normalized Ratio (INR) result and target therapeutic range; and adverse reactions or side effects associated with oral anticoagulant use (bleeding, bruising, thromboembolic events, etc.).

#### 2. Hospital Anxiety and Depression Scale (HADS)

This 14-item self-assessment screening scale has been translated and adapted into Brazilian Portuguese. It consists of 7 items for anxiety and 7 for depression, which address psychological symptoms, and yields two subscales (HADS-A and HADS-D). Each item is scored on a four-point response scale ranging from 0 to 3, and scores for each subscale can range from 0 to 21 points^(10)^. Scores were categorized as follows: 0-7, no symptoms; 8-10, mild symptoms; and 11-21, significant symptoms^(3,11)^. In the Brazilian version, Cronbach’s alpha coefficients for the anxiety and depression subscales were 0.68 and 0.77, respectively^(10)^.

#### 3. Medical Outcomes Study Social Support Survey (MOS-SSS):

This is a 19-item questionnaire developed by Sherbourne and Stewart^(8)^ to assess the extent to which individuals can count on support from others when dealing with life situations. The original instrument comprises five dimensions^(8)^; however, subsequent investigations suggested that the emotional and informational support subscales should be combined into a single dimension^(12,13)^.

Accordingly, four dimensions were analyzed: emotional/informational support (8 items), which reflects the ability of the social network to meet individual needs related to emotional problems, such as situations requiring confidentiality, encouragement in difficult times, and the presence of people who can advise, inform, and guide; tangible support (4 items), defined as the provision of practical resources and material help; affectionate support (3 items), which refers to physical expressions of love and affection; and positive social interaction (4 items), meaning having people with whom one can relax and have fun.

Participants rated each item on a five-point Likert-type scale: 1 (Never), 2 (Rarely), 3 (Sometimes), 4 (Almost always), and 5 (Always). The authors recommend calculating the mean item score for each subscale^(8)^. Higher scores indicate greater perceived support^(12,13)^. The Brazilian Portuguese validation reported Cronbach’s alpha coefficients of at least 0.83 for all dimensions^(13)^.

In this study, MOS-SSS social support scores were categorized as follows(14): tangible support, ≤ 6 points (low perception), 7-13 points (medium perception), and ≥ 14 points (high perception); affectionate support, ≤ 4 points (low perception), 5-10 points (medium perception), and ≥ 11 points (high perception); emotional/informational support, ≤ 12 points (low perception), 13-28 points (medium perception), and ≥ 29 points (high perception); and positive social interaction, ≤ 6 points (low perception), 7-13 points (medium perception), and ≥ 14 points (high perception)^(14)^.

### Data analysis and statistics

Data were entered into an Excel^®^ spreadsheet and exported to Jamovi^®^ statistical software (version 2.6). Analyses included descriptive and inferential statistics. Categorical variables were summarized as absolute and relative frequencies. Numerical variables were described using measures of central tendency and dispersion (mean, standard deviation, median, and interquartile range). Normality was assessed with the Kolmogorov-Smirnov test, which indicated a non normal distribution.

Spearman’s rank correlation coefficient was used to examine the relationships between variables. Correlation coefficients were interpreted as follows: ≤ 0.30, weak; between 0.40 and 0.60, moderate; and > 0.70, strong^(15)^. The level of statistical significance was set at *p* < 0.05.

## RESULTS

A total of 102 patients with AF participated in the study. With respect to sociodemographic characteristics (Table 1), age ranged from 32 to 87 years, with a mean of 64.53 ± 11.37 years; 72 (70.6%) participants lived in João Pessoa, Paraíba; 57 (55.9%) were male; 87 (85.3%) were economically inactive; 62 (60.8%) self-identified as Brown; and 58 (56.9%) were married or in a common-law marriage. Mean educational attainment was 6.23 ± 4.59 years, and 61 (59.8%) had a family income of up to one minimum wage.

**Table 1:** Sociodemographic characteristics of patients with atrial fibrillation, João Pessoa, Paraíba, Brazil, 2024 (N = 102)

Sociodemographic characteristics	Mean ± SD or n (%)
**Age (years)**	64.53 ± 11.36
**Place of residence**	
João Pessoa	72 (70.6)
Other municipalities	30 (29.4)
**Sex**	
Male	57 (55.9)
Female	45 (44.1)
**Employment status**	
Economically inactive	87 (85.3)
Economically active	15 (14.7)
**Skin color**	
Brown	62 (60.7)
White	22 (21.6)
Black	16 (15.7)
Indigenous	2 (2.0)
**Marital status**	
Married/common-law marriage	58 (56.9)
Never married	16 (15.7)
Widowed	15 (14.7)
Divorced/separated	13 (12.7)
**Educational attainment (years)**	6.23 ± 4.59
**Family income**	
1 minimum wage	61 (59.8)
2 minimum wages	30 (29.4)
3 minimum wages	6 (5.9)
> 4 minimum wages	5 (4.9)

*SD - standard deviation; Minimum wage - BRL 1,320.00 at the time of the study.*

Regarding clinical characteristics, the duration of treatment for AF was 6.72 ± 5.98 years, and the number of comorbid conditions ranged from 0 to 10, with a mean of 3.76 ± 1.45 per patient. In total, 80 (33.2%) participants had hypertension, 87 (35.7%) were taking beta-blockers, and 41 (40.2%) were taking warfarin as an oral anticoagulant. The mean INR was 2.43 ± 0.72. The most frequent adverse reactions to oral anticoagulant use were bleeding, reported by 14 (6.9%) participants, and bruising, reported by 13 (6.4%). The number of medications taken ranged from 2 to 11, with a mean of 6.23 ± 1.99.

Table 2 shows the scores for anxiety and depressive symptoms. Among participants, 60.8% had anxiety symptoms (mild symptoms: 32.4%; significant symptoms: 28.4%), and 39 (38.2%) had depressive symptoms (mild symptoms: 17.6%; significant symptoms: 20.6%).

**Table 2:** Scores for anxiety and depressive symptoms, João Pessoa, Paraíba, Brazil, 2024 (N = 102)

Variables	Median (IIQ)	Min.-Max.	n (%)
**Anxiety**	8.00 (7.00-11.0)	2 - 21	
No symptoms			40 (39.2)
Mild symptoms			33 (32.4)
Significant symptoms			29 (28.4)
**Depression**	6.00 (3.00-10.0)	0-17	
No symptoms			63 (61.8)
Mild symptoms			18 (17.6)
Significant symptoms			21 (20.6)

*IIQ - intervalo interquartil; Mín - mínimo; Máx - máximo.*

Table 3 presents the categorization of responses by MOS-SSS dimension. Tangible and affectionate support showed the highest percentages of participants reporting high perceived support among the four dimensions: tangible support (90.2%), affectionate support (90.2%), emotional/informational support (76.5%), and positive social interaction (70.6%).

**Table 3:** Frequency of responses on the Medical Outcomes Study Social Support Survey, João Pessoa, Paraíba, Brazil, 2024 (N = 102)

Dimensions	n (%)
**Tangible support**	
Low perception	2 (2.0)
Medium perception	8 (7.8)
High perception	92 (90.2)
**Affectionate support**	
Low perception	2 (2.0)
Medium perception	8 (7.8)
High perception	92 (90.2)
**Emotional/informational support**	
Low perception	3 (2.9)
Medium perception	21 (20.6)
High perception	78 (76.5)
**Positive social interaction**	
Low perception	6 (5.9)
Medium perception	24 (23.5)
High perception	72 (70.6)

Table 4 presents the correlation coefficients between anxiety and depressive symptom scores and social support. There was a weak, statistically significant negative correlation between HADS-D scores and affectionate support, emotional/informational support, and positive social interaction, indicating that higher scores in these social support dimensions were associated with lower depressive symptom scores. No statistically significant correlations were found between HADS-A scores and the social support dimensions.

**Table 4:** Correlation between anxiety and depressive symptom scores and social support, João Pessoa, Paraíba, Brazil, 2024 (N = 102)

Variables	Affectionate support	Tangible support	Emotional/informational support	Positive social interaction
Anxiety	-0.059	-0.182	-0.126	-0.055
Depression	-0.264^^*^^	-0.138	-0.398^^**^^	-0.379^^**^^

* p < 0.01;

** p < 0.001.

Table 5 shows the correlation coefficients for anxiety and depressive symptom scores, social support, and sociodemographic and clinical variables. A weak, statistically significant positive correlation was observed between age and tangible support, as well as between educational attainment and positive social interaction. A weak, statistically significant negative correlation was also found between the number of comorbid conditions and emotional/informational support. In addition, weak, statistically significant correlations were observed between family income and HADS-A scores and between the number of comorbid conditions and HADS-D scores.

**Table 5:** Correlation between anxiety and depressive symptom scores, social support, and sociodemographic and clinical variables, João Pessoa, Paraíba, Brazil, 2024 (N = 102)

Variables	Anxiety	Depression	Affectionate support	Tangible support	Emotional/ informational support	Positive social interaction
Age	-0.146	0.081	-0.01	0.202^^*^^	-0.029	-0.082
Educational attainment	0.085	0.031	0.026	-0.075	0.026	0.211^^*^^
Family income	-0.236^^**^^	-0.125	0.083	0.139	0.046	0.025
Number of comorbid conditions	0.159	0.267^^**^^	-0.111	-0.155	-0.205^^*^^	-0.159
Number of medications	0.035	0.067	-0.067	0.000	0.029	-0.105

* p < 0.05;

** p < 0.001.

## DISCUSSION

This study analyzed the relationship between anxiety and depressive symptoms and social support in patients with AF. The sociodemographic profile showed that most participants were older adults, male, married or in a common-law marriage, economically inactive, and had low educational attainment. These findings are consistent with the literature, which indicates that AF is associated with advancing age^(1,16)^ and has a higher prevalence among individuals aged 65-74 years^(17)^, men^(18,19)^, and those with low educational attainment^(20)^.

With respect to clinical characteristics, participants commonly had hypertension and were taking warfarin and beta-blockers. Our findings are in line with previous investigations that identified hypertension as the most frequent comorbid condition among patients with AF^(16,19,20)^. In a cross sectional study, beta-blockers were prescribed to 81% of participants for AF control^(17)^. In the RECALL study, 69.5% of patients were taking beta-blockers, 25.7% were using calcium channel blockers (CCBs), and 15.7% were receiving digitalis^(5)^.

In this study, the presence of anxiety and depressive symptoms was similar to that reported in previous research^(21-23)^. These findings may be explained by the role psychological symptoms play in cardiovascular diseases such as AF, through increased catecholamine release and sympathetic hyperactivity, which can lead to changes in heart rate variability^(24,25)^.

AF requires strict laboratory and dietary control, which may contribute to higher levels of anxiety and depressive symptoms. In addition, the use of oral anticoagulants such as warfarin requires more frequent clinic visits and laboratory tests to adjust therapeutic doses. In this study, a considerable proportion of participants were taking warfarin, likely because of its lower cost and free provision by the Brazilian Unified Health System (*Sistema Único de Saúde*, SUS), which may help explain our findings.

One study also identified higher levels of anxiety and depressive symptoms among participants who were taking warfarin compared with those using direct oral anticoagulants (DOACs)^(26)^. DOACs offer more predictable therapeutic effects, require less monitoring, and have fewer drug and dietary interactions, but cost remains a major barrier to their incorporation into clinical practice and needs to be overcome to reduce potential complications and adverse outcomes in this population^(27)^.

Participants reported high levels of perceived social support across all dimensions. Studies using the Medical Outcomes Study Social Support Survey in different chronic conditions have found similar results among older adults with cancer and individuals with HF^(28,29)^. A review highlighted the central role of family support in the management of chronic conditions. Spouses and partners are the main source of support when individuals face relational, physical, and emotional difficulties^(30)^.

In this study, depressive symptoms were inversely correlated with emotional/informational support, affectionate support, and positive social interaction. This finding is consistent with the literature^(30)^ and suggests that emotional and financial support, together with the help provided by family members, friends, and health professionals, are key factors that promote treatment adherence and help patients adapt to the therapeutic complexity involved in managing AF^(31)^. One study showed that negative affect was associated with a greater burden of AF symptoms, such as dizziness and palpitations^(32)^. Thus, social support plays an important protective role by buffering the impact of stressors and improving cardiovascular health in the short and long term^(33)^.

Social isolation and difficulties in social interaction increase the likelihood of depressive symptoms, which are commonly observed among patients with chronic diseases because of the need for lifestyle changes^(34,35)^. A review on factors associated with anxiety and depression in older adults highlighted that lower levels of social interaction are associated with more frequent reports of depressive symptoms^(36)^. Therefore, developing strategies that foster social interaction is essential to encourage patients’ engagement with treatment and improve QoL.

In this study, anxiety symptoms were not associated with social support. However, evidence indicates that, among older adults with AF, more severe anxiety symptoms are related to greater frailty in the physical and social domains^(37)^. In a Polish study, anxiety symptoms correlated significantly with poorer QoL^(38)^. Therefore, further studies are needed to explore potential relationships between these variables in greater depth.

The results also showed an association between the number of comorbid conditions and emotional/informational support. This accumulation of comorbid conditions is often described as multiple chronic conditions (MCCs), or multimorbidity, defined as the coexistence of two or more long-term health conditions^(39)^. Thus, recurrent AF symptoms combined with other chronic conditions can cause psychological distress and impair social functioning and daily activities. These effects are exacerbated by fragile support networks, caregiver burden related to managing medications and appointments, and difficulties in providing encouragement and understanding in stressful situations-factors that together contribute to poor adherence to prescribed therapy^(24,25)^.

The findings also showed a relationship between the number of comorbid conditions and depressive symptoms. AF is a condition whose prevalence increases with age, and older patients often have multimorbidity, which is associated with worse clinical outcomes^(2)^. Moreover, a higher comorbidity burden contributes to the pathogenesis of AF, including effects on intracardiac hemodynamics, inflammation, cerebral hypoxia, and metabolic changes^(40)^.

Living with multimorbidity can lead to persistent anxiety and concern about health, increasing the stress associated with ongoing treatment demands, uncontrolled symptoms, polypharmacy, and uncertainty about the future, thereby raising the risk of depression^(41)^. A study of individuals with MCCs showed that positive social support from spouses, children, relatives, and friends can significantly mitigate the detrimental impact of MCCs on depression^(42)^. Qualitative studies are recommended to explore this relationship further, including how depressive symptoms and social support shape the experiences of people with AF.

In this study, participants with more years of education tended to report higher levels of positive social interaction. Evidence suggests that people with higher educational attainment develop more complex and resilient neural networks. These individuals have a greater sense of control and better coping skills, which facilitates the mobilization of cognitive and emotional resources to deal with stressful situations^(43)^. Longitudinal studies are needed to examine this relationship and strengthen the evidence on this topic.

### Study limitations

This study has some limitations. First, its cross-sectional design precludes establishing cause and-effect relationships. Second, the use of a convenience sample and data collection at a single hospital may not be representative of other settings. Self-reported variables may also have been subject to social desirability and recall bias, since cognitive impairment was identified based on information in the medical records. In addition, the statistically significant correlations observed were of weak magnitude, which warrants caution when generalizing these findings to clinical practice. Future longitudinal studies should be conducted to assess how the variables investigated relate to clinical outcomes in people with AF.

### Contributions to the field

This study provides relevant evidence to advance contemporary scientific knowledge. In clinical practice, nurses and other members of the multidisciplinary team can draw on these findings to develop more tailored therapeutic plans that account for psychosocial symptoms and social support throughout treatment, thereby providing high-quality, holistic care. The results also support the development of interventions and health education strategies to improve indicators related to symptom management, treatment adherence, and QoL among individuals with AF.

## CONCLUSIONS

The findings of this study suggest that higher levels of affectionate support, emotional/informational support, and positive social interaction are associated with lower levels of depressive symptoms in patients with AF. No associations were found between anxiety symptoms and social support. Screening for these symptoms should therefore be a priority and a key responsibility for health professionals, particularly nurses, in the ongoing care of this population.

## Data Availability

The research data are available only upon request.
