# Supplementary material for: Relationship between anxiety and depressive symptoms and social support in patients with atrial fibrillation
Source: Rev Bras Enferm. 2026 Jul 31;79:e20250146. doi: 10.1590/0034-7167-2025-0146 (PMC13427021; doi:10.1590/0034-7167-2025-0146)
Supplement: Translation

# Translation: Relação entre sintomas de ansiedade, depressão e apoio social em pacientes com fibrilação atrial

**DOI:** 10.1590/0034-7167-2025-0146pt

**Authors:** Amanda da Silva Monteiro, Gustavo Carvalho de Lima Queiroz, Rayana Pereira Feitosa, José Arthur Guimarães dos Santos, João Victor Batista Cabral, Alcides Viana de Lima, Mailson Marques de Sousa

**Affiliations:** I Universidade Federal da Paraíba. João Pessoa, Paraíba, Brasil; II Universidade Federal do Rio Grande do Norte. Santa Cruz, Rio Grande do Norte, Brasil

**Keywords:** Fibrilação Atrial, Arritmia Cardíaca, Ansiedade, Depressão, Apoio Social.

## Abstract

**Objetivos:**

analisar a relação entre sintomas de ansiedade e depressão e apoio social em pacientes com fibrilação atrial.

**Métodos:**

estudo descritivo, transversal e correlacional, realizado com 102 participantes. Coletaram-se os dados usando os instrumentos Hospital Anxiety and Depression Scale e Escala de Apoio Social. Utilizou-se estatística descritiva e correlação de Spearman na análise dos dados.

**Resultados:**

a mediana dos escores de ansiedade foi de 8,0 (IIQ: 7-11); e a de depressão, 6,0 (IIQ: 3-10). Sintomas de ansiedade foram identificados em 60,8% dos participantes; e sintomas de depressão, em 38,2%. Observou-se correlação inversa entre apoio afetivo, apoio emocional/informacional e interação social positiva com os sintomas depressivos. Não houve associações entre os sintomas de ansiedade e o apoio social.

**Conclusões:**

níveis mais elevados de apoios afetivo e emocional e de interação social associam-se a menores escores de sintomas depressivos.

## INTRODUÇÃO

A fibrilação atrial (FA) é a taquiarritmia supraventricular mais frequente na prática clínica. Caracteriza-se por uma desorganização da atividade elétrica dos átrios, com descoordenação dos impulsos elétricos e perda da contração atrial eficaz^(1)^.

A incidência de casos de FA cresceu 13% nos últimos 20 anos, com 5 milhões de novos casos anuais em decorrência do envelhecimento populacional, avanços terapêuticos e maior sobrevida de pessoas com doenças crônicas^(2,3)^. Estima-se que cerca de 59 milhões de pessoas no mundo vivem com FA^(4)^. No Brasil, as estatísticas do Primeiro Registro Cardiovascular Brasileiro de Fibrilação Atrial (RECALL) apontam que a doença afeta cerca de 1,5 milhão de indivíduos^(5)^, com maior prevalência em idosos^(1)^, sendo considerada um problema de saúde pública.

A abordagem terapêutica de uma pessoa com diagnóstico de FA envolve mudanças no estilo de vida, reconhecimento de sintomas, manejo de comorbidades e adesão à terapêutica medicamentosa, visando mitigar eventos adversos, como acidente vascular encefálico (AVE), insuficiência cardíaca (IC), depressão e custos econômicos para os serviços de saúde^(3)^.

Os sintomas de ansiedade e depressão são observados em pessoas com FA^(1,3)^. Essas condições psicológicas provocam pior percepção da qualidade de vida (QV) e podem influenciar os desfechos clínicos, gerando um ciclo vicioso que frequentemente culmina em sofrimento diante de limitações de atividades de vida diária, má adesão terapêutica e aumento de hospitalizações evitáveis^(6,7)^. Nesse contexto, o apoio social constitui uma estratégia fundamental no manejo desses indivíduos, pois contribui para o enfrentamento emocional da doença, favorece a adesão ao tratamento e promove maior bem-estar físico e psicológico^(1)^.

O apoio social é conceituado como a disponibilidade de recursos oferecidos por outras pessoas, especialmente familiares, amigos e profissionais de saúde^(8)^. Esse conceito abrange o quanto o indivíduo está integrado socialmente, bem como o grau em que os relacionamentos interpessoais podem ajudar os indivíduos em situações críticas ou de readaptação. Logo, o apoio social atua como um fator positivo no enfrentamento da FA, favorecendo a adoção e manutenção de comportamentos saudáveis de estilo de vida^(3)^.

Diretrizes recentes da Sociedade Europeia de Cardiologia recomendam que a avaliação de sintomas relacionados à FA, como ansiedade e depressão, seja incorporada à prática clínica na oferta de um cuidado abrangente^(2,6)^. Portanto, investigar a relação entre ansiedade, depressão e apoio social em pacientes com FA mostra-se relevante para o aprimoramento da prática clínica dos enfermeiros, uma vez que esses profissionais são responsáveis pelo acompanhamento e implementação de ações de cuidado longitudinal. Além disso, necessitam desenvolver competências para identificar e manejar fatores de risco que possam contribuir para a progressão da FA.

Na América Latina, as pesquisas sobre a temática são incipientes. Até o momento, nenhum estudo foi encontrado na Região do Nordeste do Brasil, o que justifica a realização desta pesquisa e de outras futuras. Diante disso, questiona-se: Qual é a relação entre sintomas de ansiedade e depressão e apoio social em pacientes com FA?

## OBJETIVOS

Analisar a relação entre sintomas de ansiedade e depressão e apoio social em pacientes com fibrilação atrial.

## MÉTODOS

### Aspectos éticos

Esta pesquisa atendeu aos preceitos éticos da Resolução nº 466/2012 do Conselho Nacional de Saúde do Brasil e obteve aprovação do Comitê de Ética em Pesquisa. Os participantes formalizaram o consentimento por meio da assinatura do Termo de Consentimento Livre Esclarecido (TCLE), em duas vias.

### Desenho, período e local do estudo

Estudo descritivo, transversal e correlacional. As recomendações do *Strengthening the Reporting of Observational Studies in Epidemiology* (STROBE) foram seguidas. A pesquisa foi desenvolvida no ambulatório de cardiologia do Hospital Universitário Lauro Wanderley (HULW), localizado no município de João Pessoa/PB, Brasil, entre setembro de 2023 e junho de 2024. Trata se de um hospital de ensino, de grande porte, vinculado à Universidade Federal da Paraíba (UFPB) e ao Sistema Único de Saúde (SUS) e gerenciado pela Empresa Brasileira de Serviços Hospitalares (EBSERH).

### População e amostra; critérios de inclusão e exclusão

A população considerada foi de 135 pacientes com FA em *follow-up* na instituição. O cálculo do tamanho amostral foi realizado por meio do *software* OpenEpi (versão 3.01), baseado em dados de estudo prévio^(9)^. Identificou-se prevalência de 34,9% de sintomas de ansiedade, com nível de confiança de 95% e erro amostral de 5%. Dessa forma, foi determinada uma amostra mínima de 98 participantes.

Inicialmente, realizaram-se consultas prévias aos prontuários dos pacientes em *follow-up* para triagem dos participantes potencialmente elegíveis, de acordo com os critérios de inclusão. Após isso, os pacientes foram convidados a participar da pesquisa.

Foram incluídas pessoas com diagnóstico clínico de FA, com idade ≥ 18 anos e em uso de anticoagulação oral crônica. Excluíram-se pacientes em tratamento de ansiedade e depressão ou em uso de medicamentos ansiolíticos e/ou antidepressivos, bem como aqueles com déficit cognitivo confirmados em prontuário.

### Protocolo do estudo

Dois graduandos de Enfermagem devidamente capacitados coletaram os dados por meio de entrevistas individuais realizadas em ambiente reservado, com duração média de 40 minutos, utilizando-se os três instrumentos descritos a seguir.

#### 1. Formulário de caracterização sociodemográfica e clínica dos participantes

Trata-se de um instrumento elaborado pelos autores. Contém as seguintes variáveis: sexo (masculino ou feminino), idade (anos completos), procedência (João Pessoa/PB ou outros municípios), cor da pele (branca, parda, negra ou indígena), escolaridade (anos de estudo completos), situação de trabalho (ativo ou inativo), renda familiar (em reais), situação conjugal (solteiro, união estável/casado, viúvo, divorciado/separado), comorbidades associadas à FA, terapêutica medicamentosa em uso, dose semanal de anticoagulante (em mg), tempo de tratamento (em anos), último resultado da *International Normalized Ratio* (INR), faixa terapêutica ideal e reações adversas e/ou efeitos colaterais associados ao uso de anticoagulante (sangramentos, hematomas, evento tromboembólico etc.).

#### 2. *Hospital Anxiety and Depression Scale (HADS)*

Esta é uma escala de triagem de autorrelato, traduzida e adaptada para o português. É composta por 14 questões (7 para ansiedade e 7 para depressão), que abordam sintomas psicológicos. Tem opções de resposta de quatro pontos que apresentam valores de 0 a 3, cuja soma pode variar de 0 a 21 pontos para cada um dos transtornos psicológicos avaliados^(10)^. As pontuações foram categorizadas da seguinte forma: 0 a 7 - sem sintomas; 8 a 10 - sintomas moderados; 11 a 21 - sintomas significativos^(3,11)^. Na versão brasileira, as subescalas de ansiedade e de depressão apresentaram coeficientes de consistência interna de 0,68 e 0,77, respectivamente^(10)^.

#### 3. Escala de Apoio Social do *Medical Outcomes Study* (MOS-SSS):

É um questionário composto por 19 itens, desenvolvido por Sherbourne e Stewart^(8)^, para analisar em que medida o indivíduo possui o apoio de outras pessoas para enfrentar situações da vida. Originalmente, o instrumento engloba cinco dimensões^(8)^; contudo, investigações sugeriram que as subescalas de apoio emocional e informacional deveriam ser agrupadas em uma mesma dimensão^(12,13)^.

Nesse sentido, foram analisadas quatro dimensões: apoio emocional/informacional (8 itens) - habilidade da rede social em satisfazer as necessidades individuais em relação a problemas emocionais, como situações que exijam sigilo, encorajamento em momentos difíceis e presença de pessoas que aconselhem, informem e orientem; apoio material (4 itens) - provisão de recursos práticos e ajuda material; apoio afetivo (3 itens) - demonstrações físicas de amor e afeto; interação social positiva (4 itens) - contar com pessoas com quem se possa relaxar e divertir.

Os participantes indicaram as respostas em uma escala do tipo Likert de cinco pontos: 1 (Nunca); 2 (Raramente); 3 (Às vezes); 4 (Quase sempre); e 5 (Sempre). Os autores recomendam o cálculo da pontuação média dos itens para cada subescala^(8)^. Pontuações mais altas indicam melhor percepção de apoio^(12,13)^. A validação para o português brasileiro apresentou coeficiente alfa de Cronbach igual ou superior a 0,83 em todas as dimensões investigadas^(13)^.

Neste estudo, a categorização dos escores do apoio social na MOS-SSS foi realizada da seguinte forma: apoio material: ≤ 6 pontos - baixa percepção; 7 a 13 pontos - média percepção; ≥ 14 pontos - alta percepção; apoio afetivo: ≤ 4 pontos - baixa percepção; 5 a 10 pontos - média percepção; ≥ 11 pontos - alta percepção; apoio emocional/informacional: ≤ 12 pontos - baixa percepção; 13 a 28 pontos - média percepção; ≥ 29 pontos - alta percepção; e interação social positiva: ≤ 6 pontos - baixa percepção; 7 a 13 pontos - média percepção; ≥ 14 pontos - alta percepção^(14)^.

### Análise dos resultados e estatística

Os dados foram organizados em planilha Excel^®^ e transportados para o *software* estatístico Jamovi^®^ (versão 2.6). A análise ocorreu por meio de estatística descritiva e inferencial. Para variáveis categóricas, foram utilizadas frequências absolutas e percentuais. As variáveis numéricas foram descritas usando-se medidas de posição e dispersão (média, desvio-padrão, mediana e intervalo interquartil). A normalidade dos dados foi avaliada pelo teste de Kolmogorov-Smirnov, que evidenciou distribuição de dados não paramétrica.

A análise do coeficiente de correlação de Spearman foi utilizada para examinar as relações existentes entre as variáveis. Para medir a força dos coeficientes de correlação, considerou-se a seguinte classificação: ≤ 0,30 - fraca magnitude; entre 0,40 e 0,60 - moderada magnitude; > 0,70 - forte magnitude^(15)^. O nível de significância estatística adotado foi de *p* < 0,05.

## RESULTADOS

Participaram do estudo 102 pacientes com FA. Quanto aos dados sociodemográficos (Tabela 1), a idade variou de 32 a 87 anos, com média de 64,53 ± 11,37 anos; 72 (70,6%) pessoas procediam da cidade de João Pessoa/PB; 57 (55,9%) eram do sexo masculino; 87 (85,3%) estavam inativos economicamente; 62 (60,8%) autodeclararam-se pardos; e 58 (56,9%) eram casados ou viviam em união estável. A escolaridade média foi de 6,23 ± 4,59 anos, e 61 (59,8%) possuíam renda de até um salário mínimo.

**Tabela 1:** Características sociodemográficas de pacientes com fibrilação atrial, João Pessoa, Paraíba, Brasil, 2024 (N = 102)

Variáveis sociodemográficas	Média ± DP ou n (%)
**Idade (anos)**	64,53 ± 11,36
**Procedência**	
João Pessoa	72 (70,6)
Outros municípios	30 (29,4)
**Sexo**	
Masculino	57 (55,9)
Feminino	45 (44,1)
**Situação de trabalho**	
Inativo economicamente	87 (85,3)
Ativo economicamente	15 (14,7)
**Cor da pele**	
Parda	62 (60,7)
Branca	22 (21,6)
Negra	16 (15,7)
Indígena	2 (2,0)
**Situação conjugal**	
Casado(a)/união estável	58 (56,9)
Solteiro(a)	16 (15,7)
Viúvo(a)	15 (14,7)
Divorciado/separado(a)	13 (12,7)
**Escolaridade (anos)**	6,23 ± 4,59
**Renda familiar**	
1 SM	61 (59,8)
2 SM	30 (29,4)
3 SM	6 (5,9)
> 4 SM	5 (4,9)

*DP - desvio padrão; SM - salário mínimo (R$ 1.320,00, na época do estudo).*

Quanto aos aspectos clínicos, o tempo de tratamento dos pacientes com FA foi de 6,72 ± 5,98 anos, e o número de comorbidades variou de 0 a 10, com média de 3,76 ± 1,45 associadas à FA; 80 (33,2%) participantes tinham hipertensão arterial sistêmica, 87 (35,7%) faziam uso de betabloqueadores e 41 (40,2%) usavam varfarina como anticoagulante oral. O INR médio foi de 2,43 ± 0,72. No tocante às reações adversas mais frequentes associadas ao uso do anticoagulante, 14 (6,9%) tiveram sangramentos, e 13 (6,4%) apresentaram hematomas. O número de medicamentos utilizados variou de 2 a 11, com média de 6,23 ± 1,99.

A Tabela 2 apresenta os escores dos sintomas de ansiedade e depressão. Dos participantes, 60,8% apresentaram sintomas de ansiedade (grau moderado = 32,4%; nível significativo = 28,4%); e 39 (38,2%) revelaram sintomas de depressão (grau moderado = 17,6%; nível significativo = 20,6%).

**Tabela 2:** Escores dos sintomas de ansiedade e depressão, João Pessoa, Paraíba, Brasil, 2024 (N = 102)

Variáveis	Mediana (IIQ)	Mín.-Máx.	n (%)
**Ansiedade**	8,00 (7,00-11,0)	2 - 21	
Ausência de sintomas			40 (39,2)
Grau moderado			33 (32,4)
Nível significativo			29 (28,4)
**Depressão**	6,00 (3,00-10,0)	0-17	
Ausência de sintomas			63 (61,8)
Grau moderado			18 (17,6)
Nível significativo			21 (20,6)

*IIQ - intervalo interquartil; Mín - mínimo; Máx - máximo.*

A Tabela 3 apresenta a categorização das respostas conforme as dimensões. O apoio material e o apoio afetivo mostraram os maiores percentuais de alta percepção em relação às quatro dimensões propostas no instrumento: apoio material (90,2%), apoio afetivo (90,2%), apoio emocional/informacional (76,5%) e interação social positiva (70,6%).

**Tabela 3:** Frequência de respostas na Escala de Apoio Social do *Medical Outcomes Study*, João Pessoa, Paraíba, Brasil, 2024 (N = 102)

Dimensões	n (%)
**Apoio material**	
Baixa percepção	2 (2,0)
Média percepção	8 (7,8)
Alta percepção	92 (90,2)
**Apoio afetivo**	
Baixa percepção	2 (2,0)
Média percepção	8 (7,8)
Alta percepção	92 (90,2)
**Apoio emocional/informacional**	
Baixa percepção	3 (2,9)
Média percepção	21 (20,6)
Alta percepção	78 (76,5)
**Interação social positiva**	
Baixa percepção	6 (5,9)
Média percepção	24 (23,5)
Alta percepção	72 (70,6)

A Tabela 4 apresenta os resultados dos coeficientes de correlação entre os sintomas de ansiedade e depressão e o apoio social. Houve correlação negativa significante de fraca magnitude entre apoio afetivo, apoio emocional/informacional, interação social positiva e HADS-depressão, ou seja, quanto maiores os níveis de apoio social nessas dimensões, menores os sintomas de depressão. Não foram observadas associações estatisticamente significantes entre o escore HADS-ansiedade e as dimensões de apoio social.

**Tabela 4:** Correlação entre os sintomas de ansiedade e depressão e o apoio social, João Pessoa, Paraíba, Brasil, 2024 (N = 102)

Variáveis	Apoio afetivo	Apoio material	Apoio emocional/informacional	Interação social positiva
Ansiedade	-0,059	-0,182	-0,126	-0,055
Depressão	-0,264^^*^^	-0,138	-0,398^^**^^	-0,379^^**^^

* p < 0,01;

** p < 0,001.

A Tabela 5 apresenta os coeficientes de correlação entre os sintomas de ansiedade e depressão, o apoio social e as variáveis sociodemográficas e clínicas. Observou-se correlação significante positiva de fraca magnitude entre idade e apoio material, bem como entre escolaridade e a dimensão de interação social positiva. Verificou-se, ainda, correlação significante negativa, também de fraca magnitude, entre o número de comorbidades e a dimensão de apoio emocional/informacional. Além disso, houve correlação significante de fraca magnitude entre renda familiar e sintomas de ansiedade, bem como entre número de comorbidades e sintomas de depressão.

**Tabela 5:** Correlação entre sintomas de ansiedade, depressão, apoio social e variáveis sociodemográficas e clínicas, João Pessoa, Paraíba, Brasil, 2024 (N = 102)

Variáveis	Ansiedade	Depressão	Apoio afetivo	Apoio material	Apoio emocional/informacional	Interação social positiva
Idade	-0,146	0,081	-0,01	0,202^^*^^	-0,029	-0,082
Escolaridade	0,085	0,031	0,026	-0,075	0,026	0,211^^*^^
Renda familiar	-0,236^^**^^	-0,125	0,083	0,139	0,046	0,025
Número de comorbidades	0,159	0,267^^**^^	-0,111	-0,155	-0,205^^*^^	-0,159
Número de medicamentos	0,035	0,067	-0,067	0,000	0,029	-0,105

* p < 0,05;

** p < 0,001.

## DISCUSSÃO

Este estudo analisou a relação entre os sintomas de ansiedade e depressão e o apoio social em pacientes com FA. As características sociodemográficas do estudo apontaram a prevalência de idosos, homens, casados ou em união estável, com baixa escolaridade e economicamente inativos. Esses resultados estão em consonância com a literatura, em que a FA está associada ao avanço da idade^(1,16)^, com maior prevalência na faixa etária de 65-74 anos^(17)^, em indivíduos do sexo masculino^(18,19)^ e com baixa escolaridade^(20)^.

No que se refere às condições clínicas, os participantes apresentaram hipertensão arterial e usavam varfarina e betabloqueadores. Investigações anteriores são corroboradas pelos resultados deste estudo quanto às comorbidades mais comuns em pacientes com FA, entre as quais a hipertensão arterial foi a principal^(16,19,20)^. Em uma pesquisa transversal, evidenciou-se que os betabloqueadores foram prescritos para 81% dos participantes para controle da FA^(17)^. No estudo RECALL, 69,5% usavam betabloqueadores, 25,7% faziam uso de bloqueadores de canal de cálcio (BCC) e 15,7% utilizavam digitálicos^(5)^.

Neste estudo, a presença de sintomas de ansiedade e depressão foi semelhante à encontrada em pesquisas anteriores^(21-23)^. Tais achados podem ser explicados pela influência que os sintomas psicológicos exercem sobre o desenvolvimento de doenças cardiovasculares, como a FA, por meio do aumento na liberação de catecolaminas e da hiperatividade simpática, podendo ocasionar variabilidade da frequência cardíaca^(24,25)^.

A FA necessita de controle laboratorial rigoroso e dietético, de modo que favorece a prevalência de sintomas depressivos e de ansiedade. Além disso, o uso de anticoagulantes orais, como a varfarina, requer maior frequência de consultas em saúde e exames laboratoriais para ajustes de doses terapêuticas. Nesta pesquisa, uma parcela considerável dos participantes fazia uso de varfarina, possivelmente em razão do baixo custo e da dispensação gratuita pelo SUS, o que pode justificar os resultados encontrados.

Uma investigação também identificou níveis mais altos de sintomas de ansiedade e depressão em participantes que utilizavam varfarina, em comparação com os anticoagulantes orais diretos (DOACs)^(26)^. O custo dos DOACs - que apresentam maior previsibilidade de resultados, menor necessidade de monitoramento e menor interação medicamentosa e dietética - ainda é um dos fatores limitantes para sua incorporação na prática clínica, e essa barreira precisa ser superada como estratégia para reduzir potenciais complicações e desfechos adversos nessa população^(27)^.

O apoio social percebido pelos participantes evidenciou alta percepção em todas as dimensões. Estudos usando a Escala de Apoio Social em diferentes condições crônicas encontraram resultados similares em idosos com câncer e em pessoas com IC^(28,29)^. Uma revisão identificou que o apoio familiar desempenha papel fundamental no manejo de condições crônicas. Cônjuges e parceiros configuram-se como a principal fonte de suporte diante de dificuldades relacionais, físicas e emocionais^(30)^.

Neste estudo, os sintomas depressivos correlacionaram-se inversamente com o apoio emocional/informacional, apoio afetivo e interação social. Essa descoberta encontra respaldo na literatura^(30)^ e sugere que os suportes emocional e financeiro, bem como o auxílio do núcleo familiar, de amigos ou de profissionais de saúde, são fatores contributivos para promover maior adesão ao tratamento e favorecer a adaptação à complexidade terapêutica envolvida na FA^(31)^. Investigação evidenciou que o afeto negativo esteve associado a uma maior sobrecarga de sintomas de FA, como tontura e palpitações^(32)^. Assim, o apoio social é importante para mitigar eventos estressores e melhorar a saúde cardiovascular em curto e longo prazo^(33)^.

O isolamento e as dificuldades de interação social potencializam a ocorrência de sintomas depressivos, comumente observados em pacientes com doenças crônicas em razão da necessidade de mudanças no estilo de vida^(34,35)^. Um estudo de revisão sobre os fatores associados à ansiedade e depressão em idosos ressalta que, quanto menor a interação social, maior o relato de sintomas depressivos^(36)^. Logo, desenvolver estratégias que favoreçam a interação social é fundamental para estimular o engajamento terapêutico e aumentar a QV.

Nesta pesquisa, os sintomas de ansiedade não apresentaram associação com o apoio social. No entanto, há evidências de que a gravidade dos sintomas ansiosos em indivíduos idosos com FA está relacionada à maior fragilidade nas dimensões física e social^(37)^. Na Polônia, observou-se que os sintomas de ansiedade correlacionam-se significantemente com piores níveis de QV^(38)^. Portanto, novas investigações são necessárias para explorar e aprofundar possíveis relações entre as variáveis.

Os resultados destacam que o número de comorbidades teve relação com o apoio emocional/informacional. Esse acúmulo de comorbidades é frequentemente descrito como múltiplas condições crônicas (MCCs), ou multimorbidade, conceito que se refere à coexistência de duas ou mais condições de saúde de longo prazo^(39)^. Dessa forma, a presença de sintomas recorrentes de FA, associados a outras condições crônicas, pode gerar sofrimento psicológico e comprometer o funcionamento social, bem como as atividades cotidianas. Tais efeitos são agravados por redes de apoio fragilizadas, pela sobrecarga de cuidadores no manejo de medicamentos e consultas e por dificuldades em oferecer incentivo e compreensão diante de situações estressora - fatores que, em conjunto, potencializam a baixa adesão à terapêutica instituída^(24,25)^.

Os achados também evidenciaram uma relação entre o número de comorbidades e os sintomas de depressão. A FA é uma doença que aumenta com a idade, e pacientes idosos geralmente apresentam multimorbidade, que está relacionada a piores desfechos clínicos^(2)^. Além disso, o número de comorbidades contribui para amplificar a patogênese da arritmia, incluindo efeitos sobre a hemodinâmica intracardíaca, inflamação, hipóxia cerebral e alterações metabólicas^(40)^.

O convívio com multimorbidades pode causar ansiedade e preocupação constantes sobre a saúde, o que aumenta o estresse associado às demandas de tratamento contínuo, sintomas não controlados, polifarmácia e incertezas sobre o futuro, elevando o risco de depressão^(41)^. Um estudo sobre pessoas com MCC mostrou que o apoio social positivo de cônjuge, filhos, familiares e amigos reduz significativamente o efeito deletério da MCC na depressão^(42)^. Recomenda-se que estudos qualitativos abordem essa relação, incluindo a influência de sintomas depressivos e a contribuição do apoio social nas experiências de pessoas com FA.

Identificou-se que os participantes com mais anos de escolaridade tendem a ter maior interação social positiva. Entende-se que pessoas com maior escolaridade desenvolvem redes neurais mais complexas e resilientes. Tais indivíduos possuem maior senso de controle e melhores habilidades de enfrentamento, o que favorece a mobilização de recursos cognitivos e emocionais para contornar adversidades estressoras^(43)^. Sugere-se que estudos futuros longitudinais examinem essa relação para consolidar as evidências sobre o tema.

### Limitações do estudo

As limitações deste estudo incluem o delineamento transversal, que não permite estabelecer relações de causa e efeito. Outro ponto é que o uso de amostra de conveniência e a coleta de dados em um único hospital podem não refletir outros cenários de pesquisa. O autorrelato das variáveis também pode ter sido influenciado pelos vieses de desejabilidade social e de memória, uma vez que o déficit cognitivo foi avaliado com base nos registros dos prontuários clínicos. Além disso, as correlações significantes observadas apresentaram fraca magnitude, o que exige cautela na generalização dos resultados obtidos para a prática clínica. Recomenda-se que pesquisas futuras, com delineamento longitudinal, sejam conduzidas para avaliar a relação entre as variáveis investigadas nos desfechos clínicos de pessoas com FA.

### Contribuições para área

Esta pesquisa apresenta evidências relevantes para o avanço do conhecimento científico contemporâneo. No âmbito da prática clínica em saúde, enfermeiros e demais profissionais da equipe multidisciplinar poderão elaborar planos terapêuticos mais assertivos na avaliação de sintomas psicossociais e de apoio social ao longo do processo terapêutico, com o fito de oferecer uma assistência qualificada e holística. Além disso, os resultados oferecem subsídios para a proposição de intervenções e ações de educação em saúde que melhorem indicadores relacionados ao gerenciamento de sintomas, à adesão terapêutica e à QV de indivíduos com FA.

## CONCLUSÕES

Os resultados deste estudo sugerem que, quanto maiores os apoios afetivo e emocional bem como a interação social, menores são os níveis de sintomas depressivos em pacientes com FA. Não foram identificadas associações entre os sintomas de ansiedade e o apoio social. Assim, o rastreamento desses sintomas deve constituir um aspecto prioritário e de responsabilidade dos profissionais de saúde, especialmente dos enfermeiros, no cuidado contínuo dessa população.

## Data Availability

Os dados de pesquisa só estão disponíveis mediante solicitação.
